# Global DNA cytosine methylation as an evolving trait: phylogenetic signal and correlated evolution with genome size in angiosperms

**DOI:** 10.3389/fgene.2015.00004

**Published:** 2015-01-29

**Authors:** Conchita Alonso, Ricardo Pérez, Pilar Bazaga, Carlos M. Herrera

**Affiliations:** ^1^Estación Biológica de Doñana, CSICSevilla, Spain; ^2^Instituto de Investigaciones Químicas, Centro de Investigaciones Científicas Isla de La Cartuja, CSIC-USSevilla, Spain

**Keywords:** angiosperms, C-value, correlated evolution, DNA cytosine methylation, epigenetics, genome size, HPLC, phylogenetic signal

## Abstract

DNA cytosine methylation is a widespread epigenetic mechanism in eukaryotes, and plant genomes commonly are densely methylated. Genomic methylation can be associated with functional consequences such as mutational events, genomic instability or altered gene expression, but little is known on interspecific variation in global cytosine methylation in plants. In this paper, we compare global cytosine methylation estimates obtained by HPLC and use a phylogenetically-informed analytical approach to test for significance of evolutionary signatures of this trait across 54 angiosperm species in 25 families. We evaluate whether interspecific variation in global cytosine methylation is statistically related to phylogenetic distance and also whether it is evolutionarily correlated with genome size (C-value). Global cytosine methylation varied widely between species, ranging between 5.3% (*Arabidopsis*) and 39.2% (*Narcissus*). Differences between species were related to their evolutionary trajectories, as denoted by the strong phylogenetic signal underlying interspecific variation. Global cytosine methylation and genome size were evolutionarily correlated, as revealed by the significant relationship between the corresponding phylogenetically independent contrasts. On average, a ten-fold increase in genome size entailed an increase of about 10% in global cytosine methylation. Results show that global cytosine methylation is an evolving trait in angiosperms whose evolutionary trajectory is significantly linked to changes in genome size, and suggest that the evolutionary implications of epigenetic mechanisms are likely to vary between plant lineages.

## Introduction

Epigenetic regulation involves DNA methylation, histone modifications and chromatin remodeling that affect many important cell functions, including regulation of gene expression and maintenance of genomic integrity (Finnegan et al., 1998b; Bender, 2004; Grant-Downton and Dickinson, 2005; Federoff, 2012). In higher plants, methylation of cytosine residues is the chief mechanism for epigenetic modification of DNA (Finnegan et al., 1998b; Jablonka and Raz, 2009). DNA methylation involves several families of plant methyltranferases, each one introducing methyl groups at specific sequences or contributing to maintain symmetric methylation after DNA replication, and plays decisive roles in plant growth and development (Finnegan et al., 1998b, 2000). Methylated cytosines occur at variable local densities throughout genic and intergenic regions (particularly in transposable elements) of nuclear plant genomes (Cokus et al., 2008; Lister et al., 2008; Zhong et al., 2013). Within species, variations in pattern (distribution across specific sites or regions) and level (proportion of total cytosines that are methylated) of cytosine methylation may induce changes in features that are important for individual fitness, including size, flowering phenology, fecundity, inbreeding depression and response to herbivory (Sano et al., 1990; Finnegan et al., 1998a; Johannes et al., 2009; Verhoeven et al., 2010; Herrera and Bazaga, 2011; Vergeer et al., 2012). Since pattern and level of cytosine methylation often are transgenerationally heritable (Jablonka and Raz, 2009; Verhoeven et al., 2010; Herrera et al., 2013), these findings have lent considerable support to hypotheses of epigenetically-driven microevolutionary change in plant populations (Sano et al., 1990; Jablonka and Raz, 2009; Paun et al., 2010; Herrera et al., 2014).

DNA methylation has been also implicated in macroevolution through its possible effects on speciation, diversification rate and appearance of evolutionary novelty, although these suggestions have so far received limited empirical support. Methylation-mediated epigenetic mechanisms may be central to speciation through polyploidization and hybridization, two processes particularly important in plant evolution (Paun et al., 2010; Jablonka, 2013). Cytosine methylation may confer long-term selective advantage by providing an unified mechanism for setting up a variety of functions (Colot and Rossignol, 1999), and allowing evolutionary increases in gene numbers and functional complexity (Bird, 1995). Methylated cytosines mutate more frequently than unmethylated ones (Jones et al., 1992; Nachman and Crowell, 2000; Ossowski et al., 2010), hence pervasive genomic methylation may influence macroevolutionary patterns by increasing the frequency of point mutations and favoring the appearance of evolutionary novelties (McClintock, 1984; Gorelick, 2003). Alterations in pattern or level of methylation of transposable elements can influence evolutionary trajectories by inducing chromosomal rearrangements and allowing for the emergence of genetic novelty (McClintock, 1984; Feschotte and Pritham, 2007; Bonchev and Parisod, 2013). Testing these mechanistic hypotheses on the role of DNA methylation in macroevolution requires detailed comparative information on methylation patterns of specific genomic regions, a possibility currently restricted to a handful of model organisms with detailed genomic information (Zemach et al., 2010; Takuno and Gaut, 2013). Nevertheless, changes in global cytosine methylation are associated with modifications in the methylation status of specific genic and intergenic regions (Messeguer et al., 1991; Steward et al., 2002; Choi and Sano, 2007). Consequently, in absence of extensive sequence-based information on cytosine methylation patterns, the analysis of global cytosine methylation provides an initial step to evaluate the role of this epigenetic mechanism in the evolution of non-model organisms (Rozhon et al., 2008). Useful insights can be gained, for example, by investigating whether global methylation level is an evolving attribute, as implicitly assumed by proposals linking genomic methylation and evolutionary success (Bird, 1995; Colot and Rossignol, 1999; Federoff, 2012). In this paper we adopt a phylogenetically-informed approach to look for evolutionary signatures of global cytosine methylation in a sample of angiosperm species.

Interspecific variation in global cytosine methylation in plants remains unexplored, although occasional remarks indicate that it may differ substantially between species (Finnegan et al., 1998b; Bender, 2004). Published estimates of global cytosine methylation refer to few species (e.g., Wagner and Capesius, 1981; Messeguer et al., 1991), are widely scattered in the literature, and have been not examined comparatively to date. It is not known, for example, whether species differences in global cytosine methylation level reflect their distinct evolutionary histories, or whether such differences are evolutionarily correlated with concomitant changes in other attributes. Genome size is an evolving, species-specific trait in angiosperms that varies over several orders of magnitude and is correlated, among other, with life history and ecological features (Bennett and Leitch, 2012; Leitch and Leitch, 2013). A substantial part of genome size variation is caused by differences in the presence and amplification of transposable elements, particularly retrotransposons (Bennetzen et al., 1998; Grover and Wendel, 2010). Transposable elements usually are the most densely methylated regions of plant genomes (Rabinowicz et al., 2003; Federoff, 2012), and methylation-based mechanisms seem to underlie the evolution of angiosperm genome size (Bird, 1995; Federoff, 2000, 2012; Grover and Wendel, 2010), hence the evolution of global methylation levels in angiosperms might be correlated with the evolution of genome size. We analyze here an assembled data set consisting of published and unpublished estimates of global cytosine methylation for species of angiosperms, along with related information on genome size, to address the following two specific questions: (1) Is interspecific variation in global cytosine methylation phylogenetically structured?, and (2) Are global cytosine methylation and genome size evolutionarily correlated? Results support the view that, in angiosperms, global DNA cytosine methylation is an evolving, phylogenetically structured trait whose evolutionary change is related to alterations in genome size. The nature of the relationship suggests that the evolutionary significance of methylation-driven epigenetic mechanisms is likely to differ between lineages depending on genome size.

## Methods

Because of its accuracy and reproducibility, high-performance liquid chromatography (HPLC) is considered a “gold standard” method for estimating global DNA methylation (Fraga and Esteller, 2002; Lisanti et al., 2013). We used a reversed-phase HPLC technique to estimate global cytosine methylation in genomic DNA from full-grown current season leaves of 21 species of wild-growing plants collected at several field sites in southeastern Spain (electronic Supplementary Material, Table S1). Species were chosen to enhance, as far as possible, phylogenetic diversity (number of families represented), particularly when genome size information was available in public data bases (see below). Two different samples, each from a distinct individual, were analyzed per species. Total genomic DNA was extracted from dry leaf samples using Qiagen DNeasy Plant Mini Kit. A 100 ng aliquot was digested with 3 U of DNA Degradase Plus^Tm^ (Zymo Research, Irvine, CA), a nuclease mix that degrades DNA to its individual nucleoside components. Digestion was carried out in a 40 μL volume at 37°C for 3 h, and terminated by heat inactivation at 70°C for 20 min. Digested samples were stored at −20°C until analyzed. Global cytosine methylation was determined with a spectrofluorimetric detection technique modified after (Lopez Torres et al., 2011), and estimated for each sample as 100 × 5mdC/(5mdC + dC), where 5mdC and dC are the integrated areas under the peaks for 5-methyl-2′-deoxycytidine and 2′-deoxycytidine, respectively.

We screened the literature for estimates of global cytosine methylation for angiosperm species obtained by application of quantitative chromatographic methods based on HPLC techniques. Since different plant parts may differ in methylation level (Messeguer et al., 1991; Finnegan et al., 1998b), only estimates referred to leaf material were considered. In the case of experimental studies that included methylation level estimates for control and treated plants, only the values referred to control groups were taken into consideration. When multiple estimates from different strains or genotypes were reported, an average was computed. In total, 46 published estimates of global cytosine methylation level referred to 34 different species were gathered (electronic Supplementary Material, Table S1). This information and the results of our HPLC analyses for Spanish plants were combined into a single data set, and a mean figure computed for each species. One species (*Arabidopsis thaliana*) was present in the two groups of data, thus the final data set consisted of mean methylation levels for 54 species in 25 plant families (electronic Supplementary Material, Table S2).

Information on genome size of the species included in our sample was obtained from the Kew Royal Botanic Gardens Angiosperm *C*-value Database (Bennett and Leitch, 2012; last accessed 10 October 2013), which incrementally collates published information on plant *C*-values (Gregory, 2005) and is the standard source for contemporary work on genome size evolution in plants (Bennett and Leitch, 2005, 2012; Leitch and Leitch, 2013). *C*-value data, corresponding to the amount in picograms of DNA contained within a haploid nucleus, were available for 42 species in our sample (77.8% of total; electronic Supplementary Material, Table S2). When more than a single *C*-value was available for a species, the mean was used.

Evolutionary relationships between the 54 species included in the sample were inferred by constructing a phylogenetic tree using the phylomatic (Webb and Donoghue, 2005) tool bundled in Phylocom 4.2 software (Webb et al., 2008) in combination with the plant megatree R20120829 (available at https://github.com/camwebb/tree-of-trees/tree/master/megatrees; last accessed 10 October 2013). The phylogenetic tree obtained was then edited manually to eliminate polytomies (mostly in Brassicaceae and Poaceae), which were resolved by consideration of detailed family- or genus-level phylogenies available in TreeBase (http://treebase.org; last accessed 10 October 2013). All tree branch lengths were arbitrarily set to unity. The phylogenetic tree used in the analyses of phylogenetic signal and correlated evolution between cytosine methylation and genome size is presented in Figure S1 of the electronic Supplementary Material.

The relationship between phylogeny and interspecific variation in global cytosine methylation in our sample was examined by testing for the presence of a phylogenetic signal in the data, defined as “a tendency for related species to resemble each other more than they resemble species drawn at random from the tree” (Blomberg and Garland, 2002). Different indices have been proposed to test for phylogenetic signal in quantitative traits, which differ in performance, sensitivity to true underlying patterns of phylogenetic signal, robustness to phylogeny size, and degree of resolution of tree structure, but are robust to missing branch length (Münkemüller et al., 2012). In order to account for these differences, four indices possessing complementary features were applied concurrently to test for the presence of a phylogenetic signal in our cytosine methylation data set, namely Moran's *I*, Abouheif's *C*_mean_, Pagel's λ, and Blomberg's K (Münkemüller et al., 2012). Moran's *I* and Abouheif's *C*_mean_ are autocorrelation indices that are not based on an evolutionary model and are unable to provide information on the strength of the phylogenetic signal. Blomberg's K and Pagel's λ assume a Brownian Motion (BM) model of trait evolution, and for both indices values close to zero denote phylogenetic independence and a value of unity indicates a trait distribution as expected under BM. Blomberg's K and Pagel's λ can therefore be used to assess the strength, or “effect size,” of phylogenetic structuring (see Münkemüller et al., 2012 for details on the indices used, including simulation-based comparisons of performance and limitations). Computations were performed with functions in the packages adephylo (Jombart et al., 2010) and phytools (Revell, 2012) for the R environment (R Core Team et al., 2013). Statistical significance was tested in all cases by randomization with 10^5^ repetitions.

Correlated evolution between global cytosine methylation and genome size was tested in the subset of 42 species with complete data. The phylogenetically independent contrasts (PICs) method was used, which takes into account the statistical non-independence of data due to the phylogenetic relationships between species (Felsenstein, 1985; Pagel, 1999). Genome size data were log_10_-transformed for analyses. Contrasts were obtained with the pic function in the ape package (Popescu et al., 2012). Departures from the BM model underlying the PICs method (continuous traits evolve randomly in any direction and amounts of change are normally distributed) may contribute to inflate Type I error (Díaz-Uriarte and Garland, 1996). Normality of contrasts, a condition expected under BM (Paradis, 2012), was tested with the Shapiro-Wilk *W* test. Significance of the relationship between the contrasts for cytosine methylation and genome size (log_10_-transformed) was tested by fitting a linear regression through the origin and testing its significance with a permutation procedure (lmorigin function in ape package; Paradis, 2012; Popescu et al., 2012). Conventional regression diagnostics were also performed, including checks for linearity, normality of residuals and homogeneity of variance (all adjusted for phylogeny) (Freckleton, 2009).

## Results

**Figure 1:**
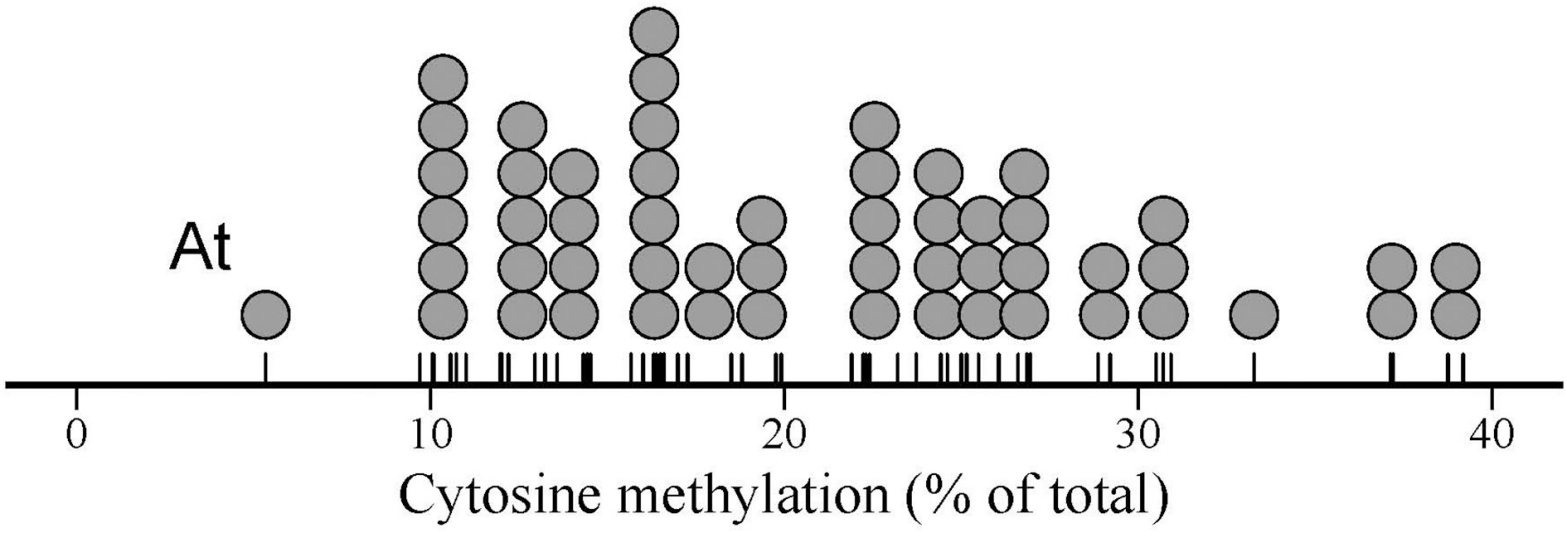
**Dot plot of global DNA cytosine methylation in the sample of 54 angiosperm species studied**. Each dot represents one species, and dot width corresponds to the maximum bin width as determined from the data using a dot-density binning algorithm. The position of data points on the axis are denoted by tick marks. “At” indicates the position of *Arabidopsis thaliana*, discussed in the text.

Species means for global cytosine methylation (%mC hereafter) varied widely in our 54-species sample (Figure 1), ranging between 5.3% (*Arabidopsis thaliana*) and 39.2% (*Narcissus nevadensis*) (interquartile range = 14.3–26.5 %; mean ± SE = 20.8 ± 1.1, *n* = 54). *Arabidopsis thaliana* stood out of the rest with a %mC value that was 1.9 standard deviations smaller than the overall sample mean (Figure 1). The broad interspecific range in %mC was not the spurious consequence of combining estimates obtained in distinct laboratories with different HPLC protocols. Mean %mC for estimates from the literature (mean ± SE = 21.9 ± 1.4 %, *n* = 46) was nearly identical to the mean value for the estimates obtained for this study (mean ± SE = 20.4 ± 1.5 %, *n* = 41) (*F*_1, 85_ = 0.53, *p* = 0.47). Within each of these two groups of data, species with two or more replicate measurements differed significantly in %mC, and interspecific variation was by far the major source of sample-wide variance (*F*_5, 12_ = 35.92, *p* << 0.001, adjusted *R*^2^ = 0.91, for literature data; *F*_19, 20_ = 53.80, *p* << 0.001, adjusted *R*^2^ = 0.96, for estimates obtained for this study). Given the contrasting sources involved in the two data groups, the close similarity in proportion of variance explained by interspecific differences further rules out methodological heterogeneity as an influential source of %mC variance in the combined sample.

**Figure 2:**
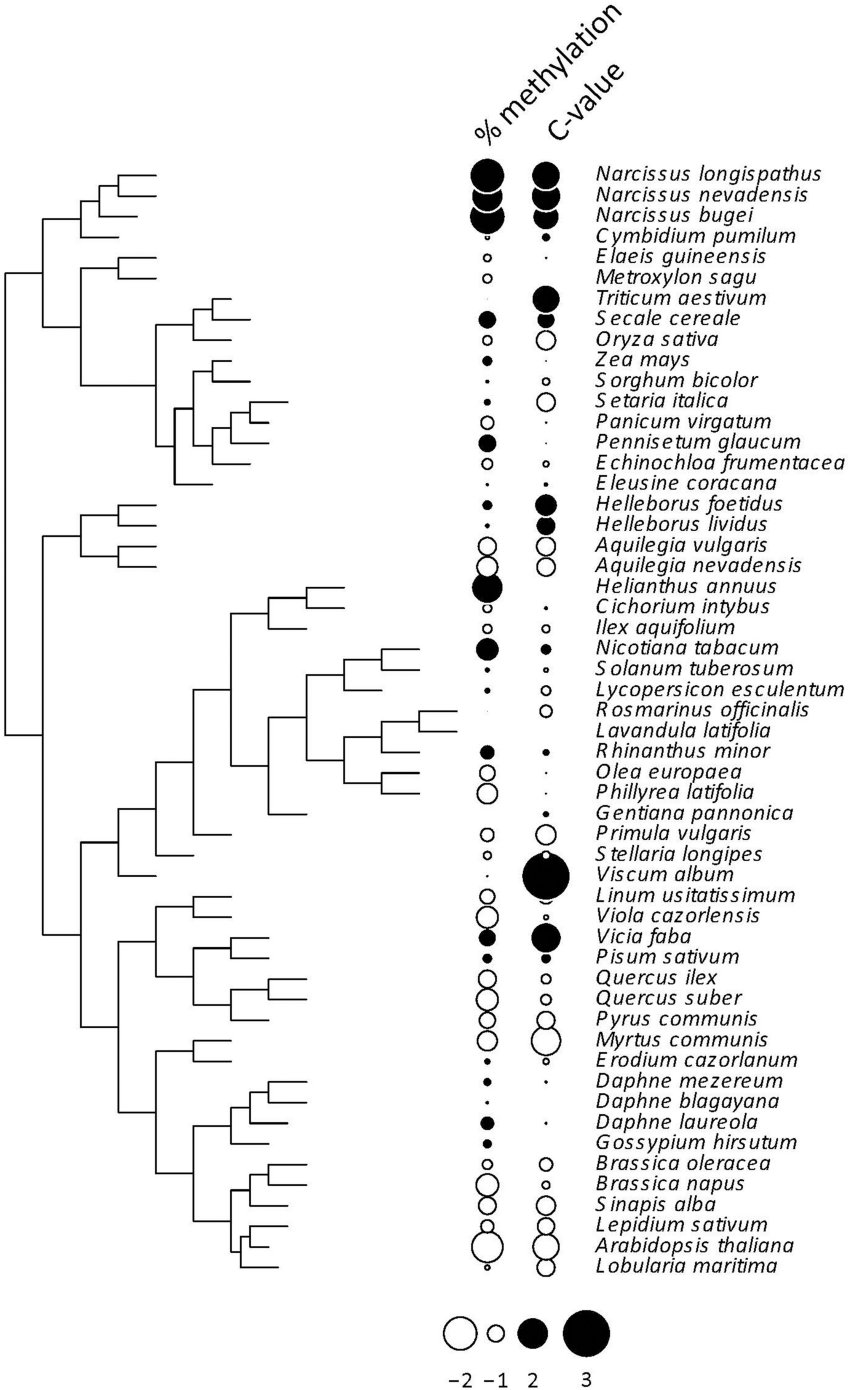
**Phylogenetic tree depicting the inferred evolutionary relationships between the 54 angiosperm species considered in this study**. Information on percent cytosine methylation and *C*-value for each species is coded as dots beside species names. The two variables were centered and scaled for the plot. See Tables S1, S2 for raw data, and Figure S1 for the distribution over the phylogenetic tree of the 12 species with missing *C*-value data.

All indices used to test for a phylogenetic signal in the %mC data set yielded statistically significant results (Table 1), thus strongly supporting the view that interspecific variation in global methylation level was phylogenetically structured in our species sample (Figure 2). Pagel's λ estimate for our data (0.877) is close to unity, the expected value for trait evolution under a pure BM model. These findings for the whole, combined sample were corroborated when literature data only were analyzed separately, and resulted much stronger when data obtained in our lab following a better hierarchical sampling of angiosperm families were analyzed separately (Table S3). Also, *C*-value exhibited a strong phylogenetic signal under BM evolutionary model (Pagel's λ = 0.999) but moderate phylogenetic autocorrelation (Table 1, Figure 2) in our moderate sample of species.

**Table 1:** **Tests for the presence of a phylogenetic signal in global DNA cytosine methylation (percent of total cytosines that are methylated) and genome size (*C*-value) in the sample of angiosperm species studied. Statistical significance was evaluated with randomization tests**.

**Phylogenetic signal index**	**Global DNA cytosine methylation (*N* = 54 species)**		***C*-value (*N* = 42 species)**	
	**Statistic**	***p*-value**	**Statistic**	***p*-value**
Moran's*I*	0.037	0.0071	−0.008	0.16
Abouheif's *C*_mean_	0.319	0.0008	0.106	0.078
Blomberg's *K*	0.392	0.0006	0.411	0.015
Pagel's λ	0.877	0.0003	0.999	0.0002

**Figure 3:**
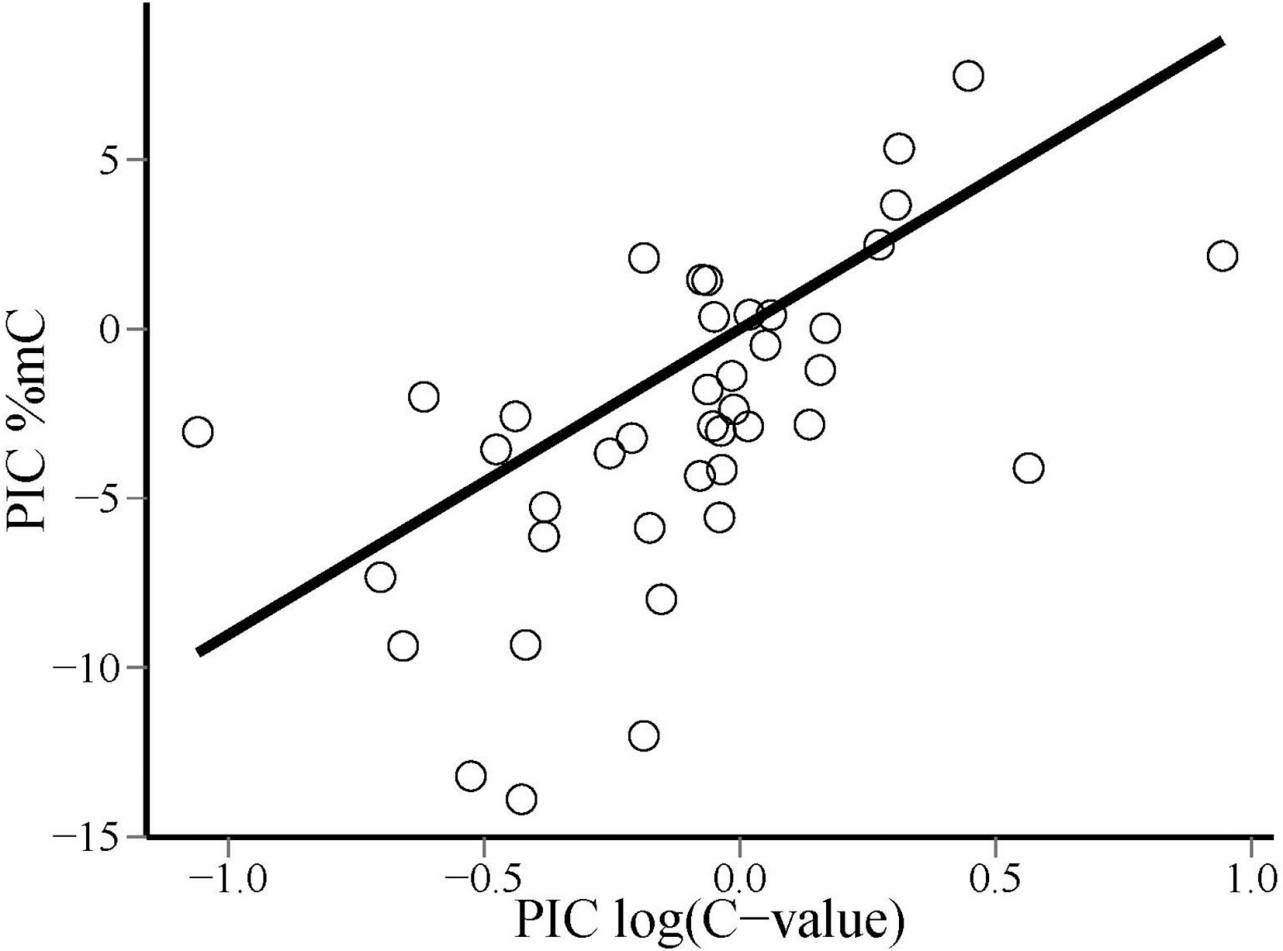
**Bivariate scatterplot of the relationship between phylogenetically independent contrasts in global DNA cytosine methylation (PIC %mC) and log_10_-transformed genome size [PIC log(*C*-value)], and least squares-fitted regression line through the origin**.

**Figure 4:**
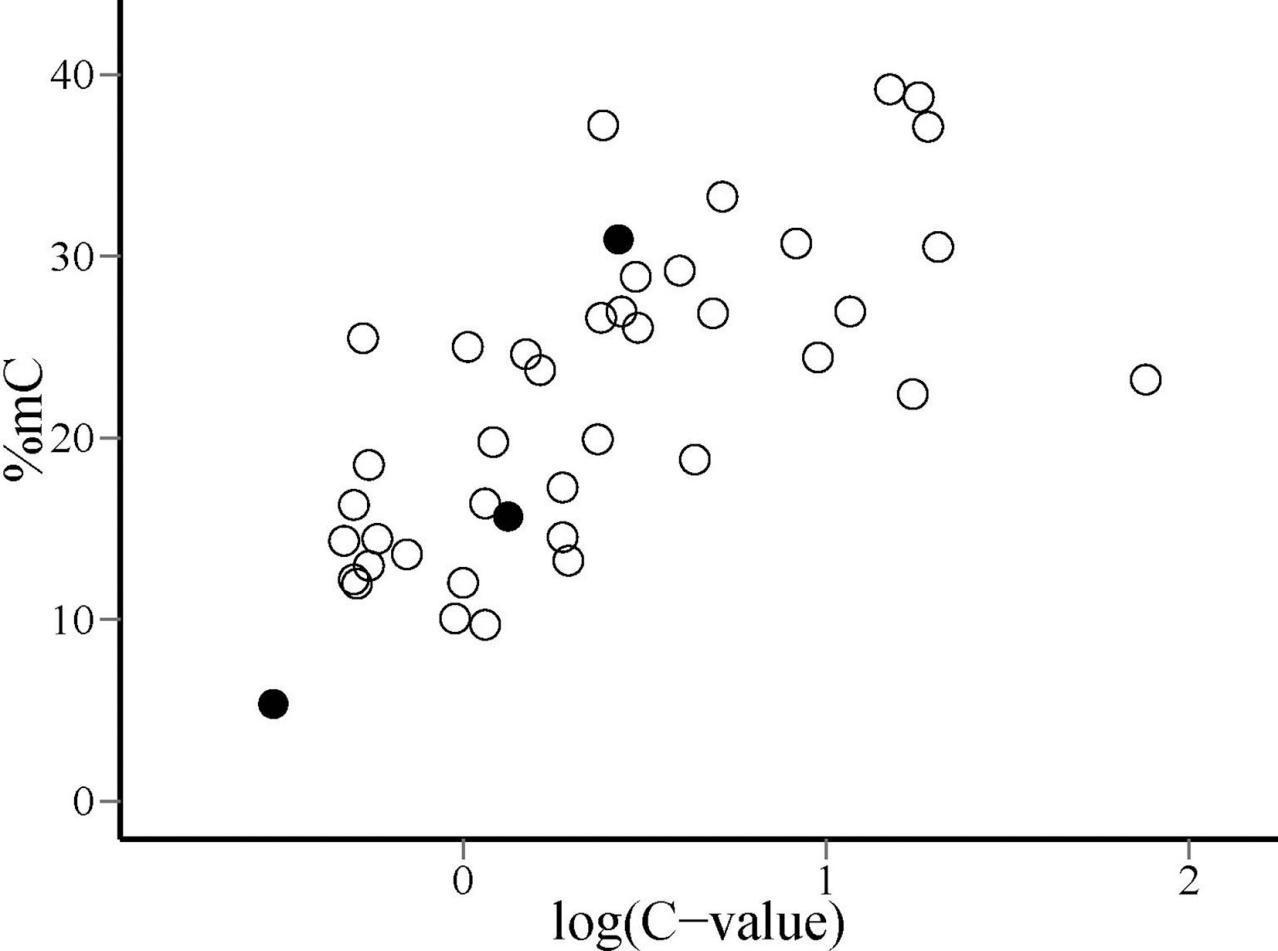
**Distribution of species on the plane defined by global DNA cytosine methylation (%mC) and log_10_-transformed genome size [log(*C*-value)]**. Filled dots indicate, from left to right, the position of *Arabidopsis thaliana*, *Oryza sativa* and *Zea mays*, discussed in the text.

Phylogenetic contrasts for cytosine methylation (PIC %mC) and (log_10_) genome size (PIC logCvalue) did not depart significantly from normality (*W* = 0.98 and 0.97, *p* = 0.54 and 0.41, respectively; Shapiro-Wilk tests). The regression through the origin of PIC %mC on PIC logCvalue was highly significant (adjusted *R*^2^ = 0.376, *F*_1, 40_ = 25.66, *p* = 0.00001). Regression residuals did not depart significantly from normality (*W* = 0.98, *p* = 0.67), and visual inspection of the plot of residuals versus predicted values did not reveal obvious departures from linearity or homocedasticity. The relationship between PIC %mC and PIC logCvalue was a direct one, revealing a correlated evolution of global cytosine methylation and (log) genome size in our species sample (Figure 3). The slope (±SE) of the fitted regression was 9.0 ± 1.8, indicating that an unity increase in log_10_ *C*-value (i.e., a 10-fold increase in genome size) was evolutionarily associated with an average increase of ~10% in global cytosine methylation. The bivariate plot of the raw, species-level data for %mC and logCvalue (uncorrected for phylogeny) reveals that the range of two orders of magnitude in *C*-value represented in our sample roughly corresponds to a range of 10–40% in %mC (Figure 4). The model plant *Arabidopsis thaliana* occupies an outlying position in the plot, with a genome simultaneously characterized by its small size and low methylation level in comparison to the rest of species considered.

## Discussion

Although global cytosine methylation measurements do not provide information on the genomic positions at which methylation occurs, it is still a valuable parameter because genome-wide methylation level can be associated with functional consequences such as mutational events, genomic instability, altered gene expression or chromosomal rearrangements (McClintock, 1984; Steward et al., 2002; Feschotte and Pritham, 2007; Rozhon et al., 2008; Bonchev and Parisod, 2013). On average, species of angiosperms considered in this study had ~20% of their genomic cytosines methylated. There was, however, considerable spread around this mean value, a result that confirms earlier suggestions of interspecific variability (Wagner and Capesius, 1981; Messeguer et al., 1991) in a larger and taxonomically diverse sample. More importantly, our results demonstrate for the first time that global cytosine methylation is an evolving trait in angiosperms and differences between species are related to their evolutionary trajectories, as revealed by the strong phylogenetic signal underlying interspecific variation.

Phylogenetic signal, the tendency for evolutionarily related species to resemble each other, is ubiquitous and has been documented for a myriad morphological, physiological, life history, behavioral and ecological traits (Blomberg et al., 2003). Simulations have shown that the ability to infer evolutionary process from the measurement of phylogenetic signal alone is limited, since different evolutionary processes can produce similar phylogenetic signal, and similar evolutionary processes can eventually lead to contrasting phylogenetic signal signatures (Revell et al., 2008). These caveats, however, apply particularly to situations where observed phylogenetic signal is low (Revell et al., 2008). In our case, both Pagel's λ and Blomberg's *K* revealed a strong phylogenetic signal in %mC data, and highly significant Moran's *I* and Abouheif's C_mean_ confirmed phylogenetic autocorrelation in %mC data. Pagel's λ is a scaling parameter for the correlations between species relative to the correlation expected under Brownian evolution, and its prevailing linear relationship with strength of Brownian motion renders it a suitable index to measure strength of phylogenetic signal (Münkemüller et al., 2012). Our λ estimate of 0.88 for %mC data and 0.99 for *C*-value are very close to the parameter's practical upper limit of unity, thus indicating a strong phylogenetic signal. In contrast to λ, Blomberg's *K* does not vary linearly with strength of Brownian motion, and its upper limit depends on the number of species in the phylogeny (Blomberg et al., 2003; Münkemüller et al., 2012). Judging from Figure 2 in Münkemüller et al. (2012), the *K*-value obtained here for %mC data and *C*-values (0.39 and 0.41, respectively) fall around the inferred upper limit for our number of species sampled, which likewise supports a strong phylogenetic signal in our %mC data set. With the due caution, therefore, we suggest that the high phylogenetic signal exhibited by interspecific variation in global cytosine methylation in our species sample should be interpreted as an indication of the traits' stochastic evolution along the hierarchical tree (Figure 2). Regarding *C*-value, our results are congruent with a handful of studies that, using one method or another, have shown a distinct phylogenetic signature of this trait in different samples (e.g., Zonneveld, 2008; Vesely et al., 2013; Kang et al., 2014).

Phylogenetic signal only describes a pattern whereby evolutionarily related organisms resemble each other more closely than unrelated ones, without further immediate implications as to the actual mechanisms that may have caused the resemblance. From a practical viewpoint, however, our demonstration of a phylogenetic signal in global cytosine methylation has two important consequences for future comparative studies on DNA methylation in angiosperms: the genome methylation level of unstudied species may be approximately predicted from knowledge of phylogenetic position and values for close relatives, and phylogenetic relationships among species should be taken into consideration in any comparative analysis relating cytosine methylation level to other species traits (Felsenstein, 1985; Pagel, 1999; Blomberg et al., 2003). From a conceptual perspective, the phylogenetic signal exhibited by global cytosine methylation provides grounds for a specific, testable hypothesis bearing on the macroevolutionary significance of epigenetic mechanisms. Given the increased mutation rates caused by the methylation of cytosines (Jones et al., 1992; Nachman and Crowell, 2000; Ossowski et al., 2010) and the crucial role of mutation in speciation (Nei, 2013), a direct correlation should be expected between global cytosine methylation and speciation rate in angiosperm lineages. Whether such evolution would occur by appearance and/or changes in the activity of different methylation systems, by threshold changes in gene body methylations or a combination of these and other potential mechanisms is yet unclear and deserves further studies (Zemach et al., 2010; Takuno and Gaut, 2013). The fact that cytosine methylation is most frequent in non-coding, intergenic regions such as transposons (Rabinowicz et al., 2003, 2005; Vaughn et al., 2007) does not conflict with this hypothesized evolutionary effect. Transposons are not exempt from the increased mutation rates caused by cytosine methylation (SanMiguel et al., 1998; Ossowski et al., 2010), and such alterations could likewise generate evolutionary novelties through modification of genome structure, gene sequences or gene regulatory functions (Banks and Federoff, 1989; Feschotte and Pritham, 2007; Martin et al., 2009; Bonchev and Parisod, 2013).

In contrast with the limited evolutionary information furnished by phylogenetic signal alone, correlations between traits in comparative studies are powerful tools to identify evolutionary processes underlying variation in a trait of interest (Harvey and Pagel, 1991; Pagel, 1999). A strong evolutionary correlation existed in our species sample between genome size and global cytosine methylation, as denoted by the high coefficient of determination (*R*^2^) of the fitted regression through the origin between PIC %mC and PIC logCvalue (Freckleton, 2009). Over the limited phylogenetic domain considered here, evolutionary changes in genome size were significantly associated with parallel modifications in global cytosine methylation as estimated by %mC. This finding is in accordance with the hypothesis postulating that methylation-based epigenetic mechanisms have played a determinant role in allowing the evolutionary increase in size and complexity of plant genomes. More specifically, it supports the proposal that “it is precisely the elaboration of epigenetic mechanisms from their prokaryotic origins as suppressors of genetic exchanges that underlies both the genome expansion and the proliferation of TEs characteristic of higher eukaryotes” (Federoff, 2012). Genome size bears only a weak relationship to gene number in plants, and transposition of repeated elements is a major cause of plant genome expansion (Bennetzen et al., 1998; SanMiguel et al., 1998). Genes do not occupy similar amounts of genomic DNA and are not packed at the same density in genomes of different sizes. Transposable elements comprise >50% of large plant genomes (e.g., maize) but <10% of small ones (e.g., *Arabidopsis*) (Bennetzen et al., 1998; Federoff, 2012). Since cytosines usually are much more densely methylated in transposons than in genes (Rabinowicz et al., 2003, 2005; Vaughn et al., 2007), then the overall %mC content of a plant genome should be directly related to its repeated sequence content (Bender, 2004). Therefore, given the known role of cytosine methylation in the control of transposon multiplication (Finnegan et al., 1998b), the correlated evolution of genome size and %mC found here supports the notion that cytosine methylation is an adaptive feature related to the evolutionary increase in plant genome arising from the multiplication of repeated elements (Federoff, 2012).

Nonlinearity of the relationship between changes in %mC and genome size suggests, however, that the evolutionary connection between the two variables is probably more complex than the simple cause-effect, proportionality relationship suggested above. The range of *C*-values represented in our species sample (0.24–76; electronic Supplementary Material, Table S2) spans ~300-fold variation, which largely overlaps the known range of variation for the angiosperms as a whole (Bennett and Leitch, 2005). The proportion of methylated cytosines increased monotonously over the sampled range of genome sizes, but linearity of the relationship between %mC and the log_10_ transform of *C*-value (Figure 3) reveals that methylation level increases at a slower pace than genome size. A ten-fold increase in genome size entailed an average increase of 10% in the proportion of methylated cytosines, which means that the probability of methylation of individual cytosines declined steadily as genomes grew larger. This pattern might be caused, among other, by a decline in the proportion of repeated elements in the genome or in the methylation density of repeated elements. Available information on the genomes of *Arabidopsis thaliana*, *Oryza sativa* and *Zea mays* tends to favor the second of these mechanisms. In these species, which exemplify the direct relationship between %mC and log(*C*-value) over a broad range of genome sizes (Figure 4), total DNA repeats account for 5, 31, and 66% of the genome, respectively (Le et al., 2000; Haberer et al., 2005), while the corresponding values for %mC are 5, 16, and 27%. These figures are compatible with the interpretation that methylation density of repeated elements tends to decline as they become proportionally more important in increasing genomes. Since the genetic activity of plant transposons depends closely on their methylation status, a decline in their *relative* methylation with increasing genome size could enhance the opportunities for genetic, phenotypic or evolutionary effects of transposable elements in plants with larger genomes (Banks and Federoff, 1989; Bonchev and Parisod, 2013). If this speculation were substantiated by future studies, then current epigenetic and epigenomic paradigms based on functional analyses of the small-genome, low-methylation, low-transposon model plant *Arabidopsis thaliana* (Schmitz and Ecker, 2012) might perhaps require some adjustments to be extrapolated to the generality of angiosperms. In any case, our results suggest the intriguing possibility that the evolutionary significance of methylation-driven, transposon-related epigenetic phenomena is likely to differ between angiosperm lineages depending on genome size.

## Conclusions

Cytosine methylation is an important epigenetic mechanism that in plants plays significant roles in the regulation of gene expression, control of genomic integrity, individual development and growth, and response to biotic and abiotic stresses (Finnegan et al., 1998b; Bender, 2004; Grant-Downton and Dickinson, 2005; Federoff, 2012). Here we show that global cytosine methylation varied widely between plant species and such variation is phylogenetically structured in angiosperms. The correlated evolution between global cytosine methylation and genome size supports the notion that cytosine methylation is an adaptive feature allowing the evolutionary increase in size and complexity of plant genomes.

## Author contributions

Conchita Alonso and Carlos M. Herrera participated in study design, sample collection, chemical and statistical analysis, and manuscript preparation. Ricardo Pérez developed the HPLC method and ran all the samples. Pilar Bazaga prepared DNA samples and enzymatic digestions, and participated in method refinement and sample processing. Carlos M. Herrera conceived the study and obtained funding. All authors read and approved the final manuscript.

### Conflict of interest statement

The authors declare that the research was conducted in the absence of any commercial or financial relationships that could be construed as a potential conflict of interest.
