# Supplementary material for: Global DNA cytosine methylation as an evolving trait: phylogenetic signal and correlated evolution with genome size in angiosperms
Source: Front Genet. 2015 Jan 29;6:4. doi: 10.3389/fgene.2015.00004 (PMC4310347; doi:10.3389/fgene.2015.00004)
Supplement: Supplementary file 1 [file Table1.DOC]

Table S1. The raw data for this study, consisting of 87 estimates of genome-wide, global DNA cytosine methylation from 54 angiosperm species obtained with chromatograhic methods. For the present study, samples were full-grown current season leaves from individual plants, collected at their natural populations during the flowering season.

| Species | % Methylated cytosines | Reference | Remarks |
| --- | --- | --- | --- |
| *Aquilegia nevadensis* | 10.56 | Present study |  |
| *Aquilegia vulgaris* | 11.33 | Present study |  |
| *Aquilegia vulgaris* | 12.59 | Present study |  |
| *Arabidopsis thaliana* | 6.65 | Kakutani et al. (1999) |  |
| *Arabidopsis thaliana* | 4.60 | Leutwiler et al. (1984) |  |
| *Arabidopsis thaliana* | 3.42 | Present study |  |
| *Arabidopsis thaliana* | 4.59 | Present study |  |
| *Arabidopsis thaliana* | 7.46 | Rozhon et al. (2008) | Leaves, mean for Zh and C2S1 |
| *Brassica napus* | 9.70 | Vanyushin & Belozerskii (1959) | As *Brassica oleifera*. Cited by Leutwiler et al. (1984). |
| *Brassica oleracea* | 16.00 | Salmon et al. (2008) |  |
| *Cichorium intybus* | 16.60 | Demeulemeester et al. (1999) |  |
| *Cymbidium pumilum* | 18.80 | Wagner & Capesius (1981) |  |
| *Daphne blagayana* | 20.72 | Present study |  |
| *Daphne blagayana* | 19.10 | Present study |  |
| *Daphne laureola* | 30.76 | Present study |  |
| *Daphne laureola* | 26.95 | Present study |  |
| *Daphne mezereum* | 25.92 | Present study |  |
| *Daphne mezereum* | 26.18 | Present study |  |
| *Echinochloa frumentacea* | 15.66 | Kumar et al. (1990) |  |
| *Elaeis guineensis* | 17.26 | Kubis et al. (2003) |  |
| *Eleusine coracana* | 23.72 | Kumar et al. (1990) |  |
| *Erodium cazorlanum* | 24.86 | Present study |  |
| *Erodium cazorlanum* | 25.44 | Present study |  |
| *Gentiana pannonica* | 22.30 | Fiuk et al. (2010) |  |
| *Gossypium hirsutum* | 26.60 | Messeguer et al. (1991) |  |
| *Helianthus annuus* | 37.20 | Messeguer et al. (1991) |  |
| *Helleborus foetidus* | 26.61 | Present study |  |
| *Helleborus foetidus* | 27.29 | Present study |  |
| *Helleborus lividus* | 25.36 | Present study |  |
| *Helleborus lividus* | 23.47 | Present study |  |
| *Ilex aquifolium* | 15.35 | Present study |  |
| *Ilex aquifolium* | 17.40 | Present study |  |
| *Lavandula latifolia* | 23.58 | Present study |  |
| *Lavandula latifolia* | 20.21 | Present study |  |
| *Lepidium sativum* | 14.43 | Yanez Barrientos et al. (2013) |  |
| *Linum usitatissimum* | 13.58 | Fieldes et al. (2005) | Mean of values in Table 3 |
| *Lobularia maritima* | 18.50 | Wagner & Capesius (1981) |  |
| *Lycopersicon esculentum* | 25.00 | Messeguer et al. (1991) |  |
| *Metroxylon sagu* | 16.50 | Novero et al. (2012) | Mean of spiny and non-spiny plants |
| *Myrtus communis* | 11.00 | Parra et al. (2001) |  |
| *Narcissus bugei* | 41.41 | Present study |  |
| *Narcissus bugei* | 36.95 | Present study |  |
| *Narcissus longispathus* | 39.51 | Present study |  |
| *Narcissus longispathus* | 37.99 | Present study |  |
| *Narcissus nevadensis* | 39.09 | Present study |  |
| *Narcissus nevadensis* | 35.14 | Present study |  |
| *Nicotiana tabacum* | 37.30 | Matassi et al. (1992) |  |
| *Nicotiana tabacum* | 27.80 | Messeguer et al. (1991) |  |
| *Nicotiana tabacum* | 34.15 | Rozhon et al. (2008) |  |
| *Nicotiana tabacum* | 34.50 | Rozhon et al. (2008) |  |
| *Nicotiana tabacum* | 32.60 | Wagner & Capesius (1981) |  |
| *Olea europaea* | 11.85 | Present study |  |
| *Olea europaea* | 14.60 | Present study |  |
| *Oryza sativa* | 18.60 | Messeguer et al. (1991) |  |
| *Oryza sativa* | 14.00 | Sano et al. (1990) |  |
| *Panicum virgatum* | 14.52 | Kumar et al. (1990) | As *Panicum sumatrense* |
| *Pennisetum glaucum* | 30.92 | Kumar et al. (1990) |  |
| *Phillyrea latifolia* | 13.43 | Present study |  |
| *Phillyrea latifolia* | 8.02 | Present study |  |
| *Pisum sativum* | 30.50 | Matassi et al. (1992) |  |
| *Pisum sativum* | 23.20 | Wagner & Capesius (1981) |  |
| *Primula vulgaris* | 15.13 | Present study |  |
| *Primula vulgaris* | 13.50 | Present study |  |
| *Pyrus communis* | 12.95 | Michalak et al. (2013) | Seedlings from control seeds |
| *Quercus ilex* | 11.03 | Present study |  |
| *Quercus ilex* | 12.99 | Present study |  |
| *Quercus suber* | 10.52 | Present study |  |
| *Quercus suber* | 9.58 | Present study |  |
| *Rhinanthus minor* | 29.20 | Wagner & Capesius (1981) |  |
| *Rosmarinus officinalis* | 24.76 | Present study |  |
| *Rosmarinus officinalis* | 19.68 | Present study |  |
| *Secale cereale* | 28.40 | Matassi et al. (1992) |  |
| *Secale cereale* | 33.00 | Thomas & Sherratt (1956) | Cited by Leutwiler et al. (1984) |
| *Setaria italica* | 25.48 | Kumar et al. (1990) |  |
| *Sinapis alba* | 12.20 | Wagner & Capesius (1981) |  |
| *Solanum tuberosum* | 24.60 | Messeguer et al. (1991) |  |
| *Sorghum bicolor* | 19.76 | Kumar et al. (1990) |  |
| *Stellaria longipes* | 16.99 | Cai & Chinnappa (1999) |  |
| *Triticum aestivum* | 22.40 | Wagner & Capesius (1981) |  |
| *Vicia faba* | 30.50 | Wagner & Capesius (1981) |  |
| *Viola cazorlensis* | 11.42 | Present study |  |
| *Viola cazorlensis* | 8.76 | Present study |  |
| *Viscum album* | 23.20 | Wagner & Capesius (1981) |  |
| *Zea mays* | 28.60 | Matassi et al. (1992) |  |
| *Zea mays* | 26.70 | Messeguer et al. (1991) |  |
| *Zea mays* | 24.80 | Papa et al. (2001) |  |
| *Zea mays* | 27.70 | Tsaftaris & Polidoros (2000) |  |

**References**

Cai Q, Chinnappa CC. 1999. Cytosine methylation levels in the genome of *Stellaria longipes*. *Journal of Biosciences* **24**, 27–33.

Demeulemeester MAC, Van Stallen N, De Proft MP. 1999. Degree of DNA methylation in chicory (*Cichorium intybus* L.): influence of plant age and vernalization. *Plant Science* **142**, 101–108.

Fieldes MA, Schaeffer SM, Krech MJ, Brown JCL. 2005. DNA hypomethylation in 5-azacytidine-induced early-flowering lines of flax. *Theoretical and Applied Genetics* **111**, 136–149.

Fiuk A, Bednarek PT, Rybczynski JJ. 2010. Flow cytometry, HPLC-RP, and metAFLP analyses to assess genetic variability in somatic embryo-derived plantlets of *Gentiana pannonica* Scop. *Plant Molecular Biology Reporter* **28**, 413–420.

Kakutani T, Munakata K, Richards EJ, Hirochika H. 1999. Meiotically and mitotically stable inheritance of DNA hypomethylation induced by ddm1 mutation of *Arabidopsis thaliana*. *Genetics* **151**, 831–838.

Kubis SE, Castilho A, Vershinin AV, Heslop-Harrison JS. 2003. Retroelements, transposons and methylation status in the genome of oil palm (*Elaeis guineensis*) and the relationship to somaclonal variation. *Plant Molecular Biology* **52**, 69–79.

Kumar LS, Hendre RR, Ranjekar PK. 1990. 5-Methylcytosine content and methylation status in six millet DNAs. *Journal of Biosciences* **15**, 47–52.

Leutwiler LS, Houghevans BR, Meyerowitz EM. 1984. The DNA of *Arabidopsis thaliana*. *Molecular and General Genetics* **194**, 15–23.

Matassi G, Melis R, Kuo KC, Macaya G, Gehrke CW, Bernardi G. 1992. Large-scale methylation patterns in the nuclear genomes of plants. *Gene* **122**, 239–245.

Messeguer R, Ganal MW, Steffens JC, Tanksley SD. 1991. Characterization of the level, target sites and inheritance of cytosine methylation in tomato nuclear DNA. *Plant Molecular Biology* **16**, 753–770.

Michalak M, Barciszewska MZ, Barciszewski J, Plitta BP, Chmielarz P. 2013. Global changes in DNA methylation in seeds and seedlings of *Pyrus communis* after seed desiccation and storage. *PLoS ONE* **8**, e70693.

Novero AU, Mabras MB, Esteban HJ. 2012. Epigenetic inheritance of spine formation in sago palm (*Metroxylon sagu* Roettb). *Plant Omics Journal* **5**, 559–566.

Papa CM, Springer NM, Muszynski MG, Meeley R, Kaeppler SM. 2001. Maize chromomethylase Zea methyltransferase2 is required for CpNpG methylation. *Plant Cell* **13**, 1919–1928.

Parra R, Pastor MT, Perez-Paya E, Amo-Marco JB. 2001. Effect of in vitro shoot multiplication and somatic embryogenesis on 5-methylcytosine content in DNA of *Myrtus communis* L. *Plant Growth Regulation* **33**, 131–136.

Rozhon W, Baubec T, Mayerhofer J, Scheid OM, Jonak C. 2008. Rapid quantification of global DNA methylation by isocratic cation exchange high-performance liquid chromatography. *Analytical Biochemistry* **375**, 354–360.

Salmon A, Clotault J, Jenczewski E, Chable V, Manzanares-Dauleux MJ. 2008. *Brassica oleracea* displays a high level of DNA methylation polymorphism. *Plant Science* **174**, 61–70.

Sano H, Kamada I, Youssefian S, Katsumi M, Wabiko H. 1990. A single treatment of rice seedlings with 5-azacytidine induces heritable dwarfism and undermethylation of genomic DNA. *Molecular and General Genetics* **220**, 441–447.

Tsaftaris AS, Polidoros AN. 2000. DNA methylation and plant breeding. *Plant Breeding Reviews* **18**, 87–176.

Wagner I, Capesius I. 1981. Determination of 5-methylcytosine from plant DNA by high-performance liquid chromatography. *Biochimica et Biophysica Acta* **654**, 52–56.

Yanez Barrientos E, Wrobel K, Lopez Torres A, Gutiérrez Corona F. 2013. Application of reversed-phase high-performance liquid chromatography with fluorimetric detection for simultaneous assessment of global DNA and total RNA methylation in *Lepidium sativum*: effect of plant exposure to Cd(II) and Se(IV). *Analytical and Bioanalytical Chemistry* **405**, 2397–2404.
