# Supplementary material for: Global DNA cytosine methylation as an evolving trait: phylogenetic signal and correlated evolution with genome size in angiosperms
Source: Front Genet. 2015 Jan 29;6:4. doi: 10.3389/fgene.2015.00004 (PMC4310347; doi:10.3389/fgene.2015.00004)
Supplement: Supplementary file 2 [file Table2.DOC]

Table S2. Species means for proportion of methylated cytosines in the genome (see Table S1 for the original raw data) and genome size (C-value, in picograms) used in this study. Dashes denote missing C-value data.

| Species | % Methylated cytosines | C-value |
| --- | --- | --- |
| *Aquilegia nevadensis* | 10.56 | – |
| *Aquilegia vulgaris* | 11.96 | 0.51 |
| *Arabidopsis thaliana* | 5.34 | 0.30 |
| *Brassica napus* | 9.70 | 1.15 |
| *Brassica oleracea* | 16.00 | 0.78 |
| *Cichorium intybus* | 16.60 | – |
| *Cymbidium pumilum* | 18.80 | 4.35 |
| *Daphne blagayana* | 19.91 | 2.35 |
| *Daphne laureola* | 28.86 | 2.99 |
| *Daphne mezereum* | 26.05 | 3.03 |
| *Echinochloa frumentacea* | 15.66 | 1.33 |
| *Elaeis guineensis* | 17.26 | 1.88 |
| *Eleusine coracana* | 23.72 | 1.63 |
| *Erodium cazorlanum* | 25.15 | – |
| *Gentiana pannonica* | 22.30 | – |
| *Gossypium hirsutum* | 26.60 | 2.40 |
| *Helianthus annuus* | 37.20 | 2.43 |
| *Helleborus foetidus* | 26.95 | 11.65 |
| *Helleborus lividus* | 24.42 | 9.50 |
| *Ilex aquifolium* | 16.38 | 1.15 |
| *Lavandula latifolia* | 21.90 | – |
| *Lepidium sativum* | 14.43 | 0.58 |
| *Linum usitatissimum* | 13.58 | 0.70 |
| *Lobularia maritima* | 18.50 | – |
| *Lycopersicon esculentum* | 25.00 | 1.03 |
| *Metroxylon sagu* | 16.50 | – |
| *Myrtus communis* | 11.00 | – |
| *Narcissus bugei* | 39.18 | 15.00 |
| *Narcissus longispathus* | 38.75 | 18.00 |
| *Narcissus nevadensis* | 37.12 | 19.10 |
| *Nicotiana tabacum* | 33.27 | 5.18 |
| *Olea europaea* | 13.23 | 1.95 |
| *Oryza sativa* | 16.30 | 0.50 |
| *Panicum virgatum* | 14.52 | 1.88 |
| *Pennisetum glaucum* | 30.92 | 2.68 |
| *Phillyrea latifolia* | 10.73 | – |
| *Pisum sativum* | 26.85 | 4.88 |
| *Primula vulgaris* | 14.32 | 0.47 |
| *Pyrus communis* | 12.95 | 0.55 |
| *Quercus ilex* | 12.01 | 1.00 |
| *Quercus suber* | 10.05 | 0.95 |
| *Rhinanthus minor* | 29.20 | 3.95 |
| *Rosmarinus officinalis* | 22.22 | – |
| *Secale cereale* | 30.70 | 8.28 |
| *Setaria italica* | 25.48 | 0.53 |
| *Sinapis alba* | 12.20 | 0.50 |
| *Solanum tuberosum* | 24.60 | 1.49 |
| *Sorghum bicolor* | 19.76 | 1.21 |
| *Stellaria longipes* | 16.99 | – |
| *Triticum aestivum* | 22.40 | 17.33 |
| *Vicia faba* | 30.50 | 20.36 |
| *Viola cazorlensis* | 10.09 | – |
| *Viscum album* | 23.20 | 76.00 |
| *Zea mays* | 26.95 | 2.73 |
