# Supplementary material for: Global DNA cytosine methylation as an evolving trait: phylogenetic signal and correlated evolution with genome size in angiosperms
Source: Front Genet. 2015 Jan 29;6:4. doi: 10.3389/fgene.2015.00004 (PMC4310347; doi:10.3389/fgene.2015.00004)
Supplement: Supplementary file 3 [file Table3.DOC]

Table S3. Tests for the presence of a phylogenetic signal in global DNA cytosine methylation (percent of total cytosines that are methylated) conducted separately for the literature and unpublished data included in the study. Statistical significance was evaluated with randomization tests.

|  | Literature data (*N* = 34 species) | |  | Unpublished data (*N* = 21 species) | |
| --- | --- | --- | --- | --- | --- |
| Phylogenetic signal index | Statistic | *p*-value |  | Statistic | *p*-value |
| Moran’s *I* | 0.058 | 0.012 |  | 0.157 | < 0.0001 |
| Abouheif’s *C*_mean_ | 0.179 | 0.060 |  | 0.622 | < 0.0001 |
| Blomberg’s *K* | 0.365 | 0.12 |  | 1.359 | < 0.0001 |
| Pagel’s λ | 0.350 | 0.11 |  | 0.999 | < 0.0001 |
