# Supplementary material for: Global DNA cytosine methylation as an evolving trait: phylogenetic signal and correlated evolution with genome size in angiosperms
Source: Front Genet. 2015 Jan 29;6:4. doi: 10.3389/fgene.2015.00004 (PMC4310347; doi:10.3389/fgene.2015.00004)

Figure S1. Phylogenetic tree depicting the inferred evolutionary relationships between the 54 angiosperm species considered in this study. In red, the 12 species without C-value data (see Table S2) which were excluded from the analyses of correlated evolution.

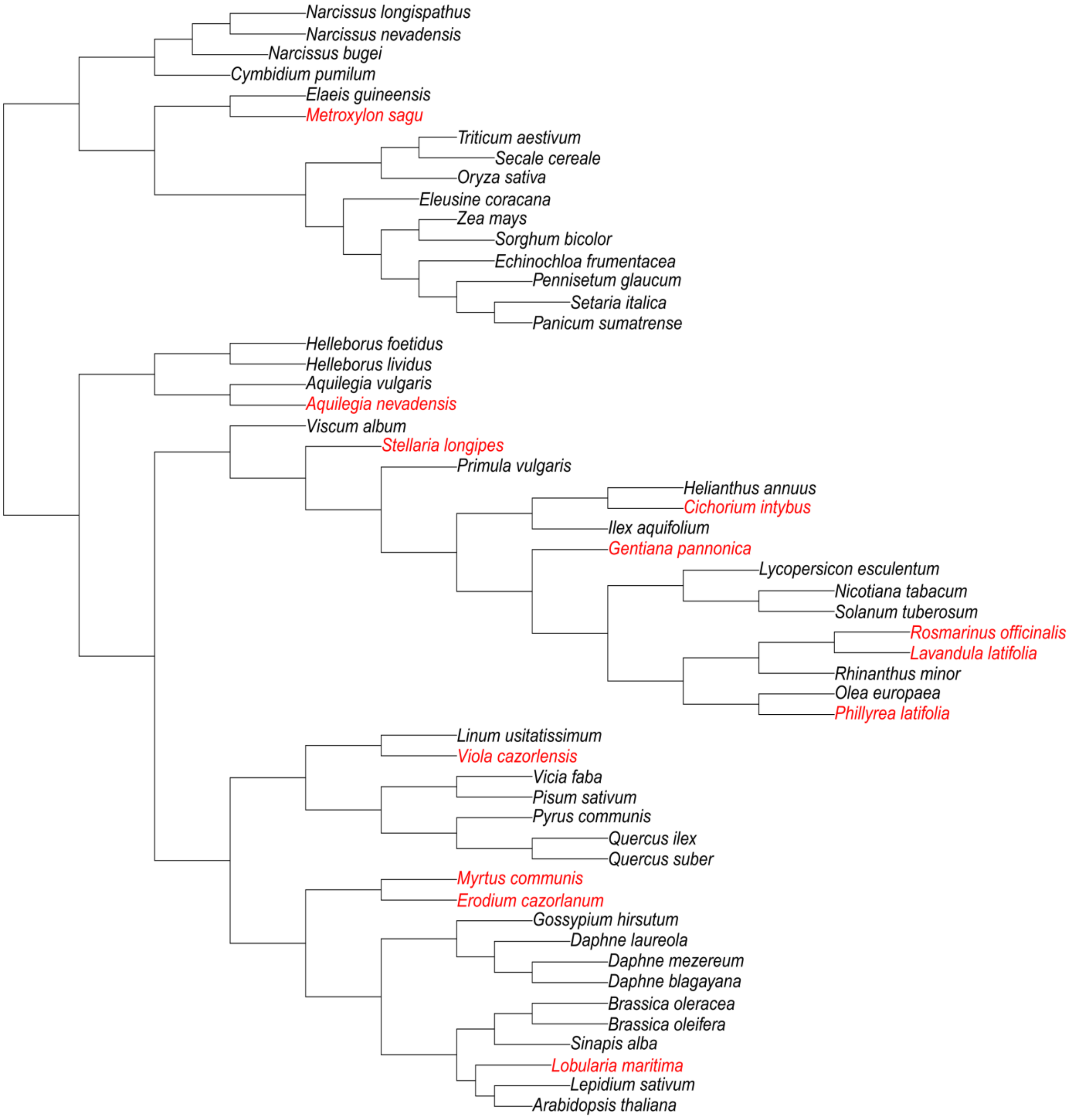

Supplement: Supplementary file 4 [file Image1.PDF]
